# Modifying a 3D-Printed Ti6Al4V Implant with Polydopamine Coating to Improve BMSCs Growth, Osteogenic Differentiation, and *In Situ* Osseointegration *In Vivo*

**DOI:** 10.3389/fbioe.2021.761911

**Published:** 2021-11-30

**Authors:** Hui Wang, Changyong Yuan, Kaili Lin, Rui Zhu, Shilei Zhang

**Affiliations:** ^1^ Department of Oral and Cranio-Maxillofacial Surgery, Shanghai Ninth People’s Hospital, College of Stomatology, Shanghai Jiao Tong University School of Medicine, National Clinical Research Center for Oral Diseases, Shanghai Key Laboratory of Stomatology and Shanghai Research Institute of Stomatology, Shanghai, China; ^2^ School of Stomatology, Xuzhou Medical University, Xuzhou, China; ^3^ Key Laboratory of Spine and Spinal Cord Injury Repair and Regeneration of Ministry of Education, Orthopaedic Department of Tongji Hospital, School of Medicine, Tongji University, Shanghai, China

**Keywords:** 3D printing, titanium alloy implants, surface modification, polydopamine, osteogenesis

## Abstract

Nowadays, 3D printing technology has been applied in dentistry to fabricate customized implants. However, the biological performance is unsatisfactory. Polydopamine (PDA) has been used to immobilize bioactive agents on implant surfaces to endow them with multiple properties, such as anti-infection and pro-osteogenesis, benefiting rapid osseointegration. Herein, we fabricated a PDA coating on a 3D-printed implant surface (3D-PDA) *via* the *in situ* polymerization method. Then the 3D-PDA implants’ pro-osteogenesis capacity and the osseointegration performance were evaluated in comparison with the 3D group. The *in vitro* results revealed that the PDA coating modification increased the hydrophilicity of the implants, promoting the improvement of the adhesion, propagation, and osteogenic differentiation of bone marrow-derived mesenchymal stem cells (BMSCs) *in vitro*. Additionally, the 3D-PDA implant improved osteointegration performance *in vivo*. The present study suggested that PDA coating might be a feasible strategy to optimize 3D-printed implant surfaces, making a preliminary research basis for the subsequent work to immobilize bioactive factors on the 3D-printed implant surface.

## 1 Introduction

Simplification of the manufacturing process and clinical procedures, and decreases in treatment time are the trends of implant dentistry. When an immediate implant technique is implemented, the implant needs to be inserted into the tooth socket immediately after tooth extraction, which can effectively preserve the height and width of the alveolar and minimize the margin bone adsorption during the healing stage of extraction socket (Koh et al., 2010). Hence, the demand for fabricating customized dental implants to precisely suit the extraction socket and obtain ideal primary stability is dramatically increasing. Fortunately, in recent years, the 3D printing technique has been applied in the orthopedic field and dentistry for its ability to fabricate customized products rapidly and effectively (van Noort 2012; Zhang et al., 2020). Based on a computer-aided design (CAD) file, 3D printing can fabricate personalized implants (Figliuzzi et al., 2012; Oladapo et al., 2021). More importantly, 3D printing could produce porous implants to make the elasticity modulus similar to natural bone, avoiding the stress shielding effect and minimizing associated bone adsorption (Sidambe 2014; Shah et al., 2016; Vijayavenkataraman et al., 2020).

Our former study found that a 3D-printed Ti6Al4V implant could obviously improve osseointegration in a rat condyle implant model compared to the implant prepared by traditional machining technology after implantation for 3 weeks. However, there was no significant difference between them in the longer term. Moreover, compared to a sand-blasting with large grit and acid-etching titanium implant (SLA), which is prevalently used in clinic, the osseointegration performance of a 3D-printed Ti6Al4V implant was inferior and not ideal (Zhang et al., 2019; Zhang et al., 2020). Therefore, it is urgent to find effective ways to overcome the unsatisfactory osseointegration performance of 3D-printed implants.

Lots of research has confirmed that the surface characteristics play essential roles in dominating implant fate (Hotchkiss et al., 2016; Zhang et al., 2021). Hence, it may be a promising strategy to make surface modifications on 3D-printed implants. So far, physical modifications, including topography, roughness, wettability, and chemical component modification have been successfully applied to the implant surface (Xia et al., 2018; Zhang et al., 2019; Liu et al., 2020; Xue et al., 2020). For example, sand-blasting, acid-etching (Zhang et al., 2020), micro-arc oxidation (Shimabukuro et al., 2019), alkali-heat treatment (Wang et al., 2018) have been proved to promote osteogenic cell lineage differentiation and implant osseointegration.

In effect, the above studies have mainly focused on providing the osteogenic property to implants for the sake of enhancing the osseointegration performance. Actually, many other factors participate in the implant osseointegration process (DAr et al., 2018; Guder et al., 2020). When the implant is placed into the human body, it can be regarded as a foreign substance, activating biological response cascades (Chen et al., 2016). Briefly, the immune cells can recognize it, and then they secrete a variety of inflammatory cytokines and chemokines, and recruit fibroblasts, endothelial cells, bone marrow mesenchymal cells (BMSCs), and other repair cells to migrate toward the implant surface to participate in the osseointegration repair process (Landen et al., 2016). Therefore, it is desired to endow an implant with multiple properties, including regulating immune cells function, promoting angiogenesis and an antimicrobial property (Chen et al., 2017; Bai et al., 2018; Zhang et al., 2021).

With the emergence and development of biochemical surface modification, bioactive agents, such as protein, peptide, growth factor, and drugs have been tentatively applied to implant surfaces. Nowadays, many techniques can immobilize the above bioactive agents, mainly classified into three kinds, protein adsorption, covalent immobilization by physical methods, or by chemical methods (Stewart et al., 2019). By assembling multiple bioactive factors on the surface, the implant would become multifunctional. Among these methods, one of the bioinspired methods, polydopamine (PDA), has attracted the attention of researchers because of its simplicity, convenience, and efficiency (Jin et al., 2020; Zeng et al., 2020; Tang et al., 2021; Wang et al., 2021; Zhuang et al., 2021).

PDA is the self-polymerized product of dopamine in an alkaline aqueous solution in the dark. Previous research confirmed that polydopamine can powerfully adhere to a large variety of material surfaces and apparently enhance hydrophilicity and biocompatibility, which is a universal method to functionalize material surfaces (Ku et al., 2010; Zeng et al., 2020; Owji et al., 2021; Tang et al., 2021). More importantly, PDA has been successfully applied to immobilize bioactive agents on implant surfaces because of numerous chemical functional groups, especially imine, amine, and catechol groups. Due to those functional groups, on the one hand, PDA can be strongly covalent with the implant surface; on the other hand, it can chelate metal ions and immobilize various bioactive factors (Lee et al., 2009). For example, Chien et al. (Chien and Tsai 2013) improved the osteogenesis of BMSCs by using polydopamine to immobilize Arg-Gly-Asp peptides, hydroxyapatite (HA), and bone morphogenic protein-2 (BMP-2) on the titanium implant surface. Poh et al. (Poh et al., 2010) utilized polydopamine to functionalize an implant surface with vascular endothelial growth factor (VEGF), which could improve human dermal microvascular endothelial cells’ (HDMECs) adhesion and multiplication, and human mesenchymal stem cells’ (hMSCs) differentiation. Using a layer by layer assemble process, Li et al. (Li et al., 2016) fabricated a hybrid coating consisting of HA, Ag nanoparticles, and chitosan on a polydopamine-modified titanium implant surface, which enhanced antimicrobial and osteogenic activity.

Overall, as mentioned before, our previous study has successfully designed and fabricated a customized 3D-printed implant. In the current research, we primarily focused on studying the biocompatibility and *in vivo* application of 3D-printed implants modified by appropriate surface modification techniques. As a bio-inspired polymer, PDA has been used as a crosslinking agent to immobilize bioactive factors on implant surfaces and modify implants biocompatibility. However, to the best of our knowledge, the research about immobilizing bioactive agents on 3D-printed Ti6Al4V implants by PDA is limited. Hence, to improve the 3D-printed Ti6Al4V implant surface, it is meaningful to explore the potential application of PDA coating *in vivo*. Therefore, in the current applied study, we tried to assemble the 3D-printed implant with a PDA coating and preliminarily studied its surface characteristics, biocompatibility, biosecurity, and osseointegration capability *in vivo*. Based on the present study results, in future work, we further intend to immobilize multiple bioactive factors on the 3D-printed implants’ surface and study its application.

## 2 Materials and Methods

### 2.1 Fabrication of Samples

The 3D-printed Ti6Al4V implants (Ø 2 mm × 4 mm) and plates (side length: 10 mm, height: 2 mm) were fabricated by an EOS laser printing system (EOS GmbH Munchen, Germany) as previously demonstrated (Wang et al., 2018). In short, the 20–50 μm Ti6Al4V alloy powers were exploited as raw materials, and then the samples were additively manufactured layer by layer (Wang et al., 2021). All the specimens were ultrasonically washed in acetone, ethanol, and distilled water, respectively, for 15 min. For the sake of fabricating the PDA coating on the specimens, the 3D-printed Ti6Al4V implants and disks were soaked in prepared dopamine solution (2 mg/ml in 10 mM Tris buffer at pH 8.5) at room temperature in the dark according to former research (Xi et al., 2009). After undergoing the polymerization reaction of dopamine monomers for 24 h, a layer of PDA was formed and deposited on the sample surfaces. Then the specimens were ultrasonically rinsed in distilled water to remove free dopamine monomers. Afterward, the samples were dried at room temperature and labeled as the 3D-PDA group. The 3D-printed Ti6Al4V implants and disks without PDA coating were named the 3D group and used as control.

### 2.2 Specimen Surface Characterization

The surface topography of the 3D-PDA and 3D groups were characterized *via* scanning electron microscopy (SEM, SU8220, Hitachi, Japan). To verify whether the PDA coating was prepared on the 3D-PDA surface, a Raman spectroscopy image was obtained using a Raman spectroscope (RW2000, Renishaw, England) at a 524 nm source wavelength. Moreover, the PDA coating thickness was detected by spectroscopic ellipsometer at the polarized angle of 70° (UVISEL, HORIBA, France). Besides, the discrepancy of hydrophilicity between the two kinds of specimens was evaluated by a surface-contact angle machine (Optical Contact Angle and interface tension meter, SL200 KS, SOLON TECH, China). The surface chemical composition of the PDA coating on the 3D-PDA group was detected by X-ray photoelectron spectroscopy (XPS, Escalab250Xi, Thermo Scientific, United States).

### 2.3 *In Vitro* Studies

#### 2.3.1 Separation and Cultivation of Bone Marrow-Derived Mesenchymal Stem Cells (BMSCs)

Sprague Dawley (SD) rats approximately 2 weeks old were purchased from the Shanghai SLAC Laboratory Animal Co. Ltd (Shanghai, China) and used in the current study. All the animal experiments and procedures were performed according to the approval and guidance of the Institutional Animal Care and Use Committee of Tongji University (Shanghai, China). The process of isolating BMSCs coincided with former research (Wang et al., 2018). Then the harvested primary cells were cultured in alpha-modified Eagle’s medium (α-MEM, Hyclone, United States) with 1% penicillin/streptomycin solution and 10% fetal bovine serum (Hyclone, United States). Cells were cultured at 37°C in a humidified atmosphere containing 5% CO_2_. Until 80–90% convergence was reached, the cells were sub-cultured and the two to four passages of BMSCs were used in the subsequent research.

#### 2.3.2 Cell Morphology and Adhesion Observation

To evaluate the form of BMSCs on the two group surfaces, 1 × 10^4^ cells were cultivated on each specimen. After culturing for 24 h, the cells were rinsed three times with phosphate buffer saline (PBS) and immobilized with 4% paraformaldehyde (PFA) overnight at 4°C. Then the samples were dehydrated with hierarchical ethanol series sequentially, freezing dried, and sprayed with gold before scanning *via* SEM.

#### 2.3.3 Cell Multiplication Activity Assay

For the cell proliferation assay, 2 × 10^4^ cells were cultured on the 3D-PDA and 3D groups. At days 1, 4 and 7, samples were rinsed with PBS three times and cultured in medium added with 10% CCK-8 (CCK-8, Beyotime, China) working solution for 3 h at 37°C in the dark. Afterward, the solution absorbance was detected at a 405 nm wavelength by utilizing a microplate reader (Biotek, United States).

#### 2.3.4 The Alkaline Phosphatase (ALP) Activity Trial

BMSCs were cultivated (2 × 10^4^/well) on the two kinds of sample surfaces to measure the ALP activity performance at days 4 and 7. For quantitative analysis of ALP activity, samples were flushed with PBS and soaked in 1% TritonX-100 (Beyotime, China). After centrifuging at 4°C (12,000 rpm × 10 min), supernatants were obtained. The ALP kit (JianCheng Bioengineering Institute, Nanjing, China) and the BCA protein trial kit (Beyotime, China) were used to test the ALP activity and total protein concentration, respectively. Ultimately, the results were computed and normalized to the total protein level.

#### 2.3.5 Isolation RNA and Quantitative Real-Time PCR (PT-PCR) Assay

BMSCs were cultivated on the different groups for 4 and 7 days to detect the osteogenesis-related gene expression level. Subsequently, the cells from each group were dissolved in Trizol reagent (Invitrogen, United States) and the total RNA was refined. Afterward, 1 μg of RNA was transcribed in reverse into cDNA by using a Prime-Script^TM^ RT reagent kit (Takara Bio, Japan). Primers utilized in this research were synthesized by Sangon Biotech (Shanghai) Co., Ltd and are listed in Table 1, including osteocalcin (OCN), osteopontin (OPN), bone sialoprotein (BSP), and collagen type 1 (COL-1). The β-actin gene was used as a reference for normalization. All RT-RCR procedures were performed on the Light Cycler 96 Real-Time PCR System (Roche, Switzerland) by utilizing the SYBR Green PCR reaction mix (Roche, Basel, Switzerland) under the instructions of the manufacturers. All the experiments were conducted in triplicate.

**TABLE 1:** Primer sequences utilized for RT-PCR.

Gene	Primer sequences (F = forward; R = reverse)
BSP	F: 5′-AGA​AAG​AGC​AGC​ACG​GTT​GAG​T-3′
	R: 5′-GAC​CCT​CGT​AGC​CTT​CAT​AGC​C-3′
OCN	F: 5′-GCC​CTG​ACT​GCA​TTC​TGC​CTC​T-3′
	R: 5′-TCA​CCA​CCT​TAC​TGC​CCT​CCT​G-3′
COL-1	F: 5′-GCC​TCC​CAG​AAC​ATC​ACC​TA-3′
	R: 5′-GCA​GGG​ACT​TCT​TGA​GGT​TG-3′
OPN	F: 5′-CCA​AGC​GTG​GAA​ACA​CAC​AGC​C-3′
	R: 5′-GGC​TTT​GGA​ACT​CGC​CTG​ACT​G-3′
β-actin	F: 5′-GTA​AAG​ACC​TCT​ATG​CCA​ACA-3′
	R: 5′-GGA​CTC​ATC​GTA​CTC​CTG​CT-3′

### 2.4 *In Vivo* Studies

#### 2.4.1 Animal Surgical Operation

In order to evaluate the osseointegration performance of the two kinds of implants, 10 SD rats weighing approximately 280 g were used in the trial. All the animal assays were authorized and performed according to the instructions of the Institutional Animal Care and Use Committee of Tongji University (Shanghai, China). The rat femoral condyle model was used in the current study and the surgical procedures were demonstrated previously (Wang et al., 2018). In short, the bilateral femoral condyles of the recipient rats were shaved and disinfected after anesthesia. Then the lateral surface of the femoral condyle was exposed, and a hole perpendicular to the long axis of the femoral condyle was prepared. Afterward, either the 3D and 3D-PDA implants were randomly inserted into the left or right femoral condyle holes. In the end, the muscle and skin were sutured in layers. After 4 weeks, the implants in the rat femoral condyles were harvested, and subsequently immobilized in 4% PFA before histological analysis.

#### 2.4.2 Histological and Histomorphometric Analyzation

Hard-tissue slicing was utilized for histological and histomorphometric observation and analysis. Briefly, the harvested samples were fixed in 4% PFA for 24 h. Then they were washed in distilled deionized water and then dehydrated in grade ethanol series from 70 to 100%. Subsequently, the samples were embedded in a light-curing one-component resin (Technovit 7200VLC, kulzers, Friedrichsdorf, Germany). After 15 h, the polymerized samples were cut longitudinally into 200-μm-thick sections with a diamond circular saw system (Exakt 300 CL, Exakt Apparatebau, Germany), and then ground and polished to around 30 μm thickness using a grinding system (Exakt 400 CS, Exakt Apparatbau, Germany).

After being stained by Van Gieson’s picrofuchsine, the sections were observed and photographed by a microscope (Olympus, Japan). And Image-Pro Plus 6.0 software was used to perform histomorphometric analysis and compute the bone-implant contact (BIC) percentage in the cancellous bone. BIC was calculated as the percentage of the length of direct contact to the total length of the implant surface.

### 2.5 Statistical Analysis

Using SPSS 22.0 statistical software, all the data were demonstrated as means ± standard deviation (SD). Independent *t*-test or paired *t*-test were conducted to compare the statistic difference between the 3D and 3D-PDA groups. The distinction was considered statistically significant at *p* < 0.05.

## 3 Results and Discussion

### 3.1 Characterization Analysis of Prepared Samples

**FIGURE 1:**
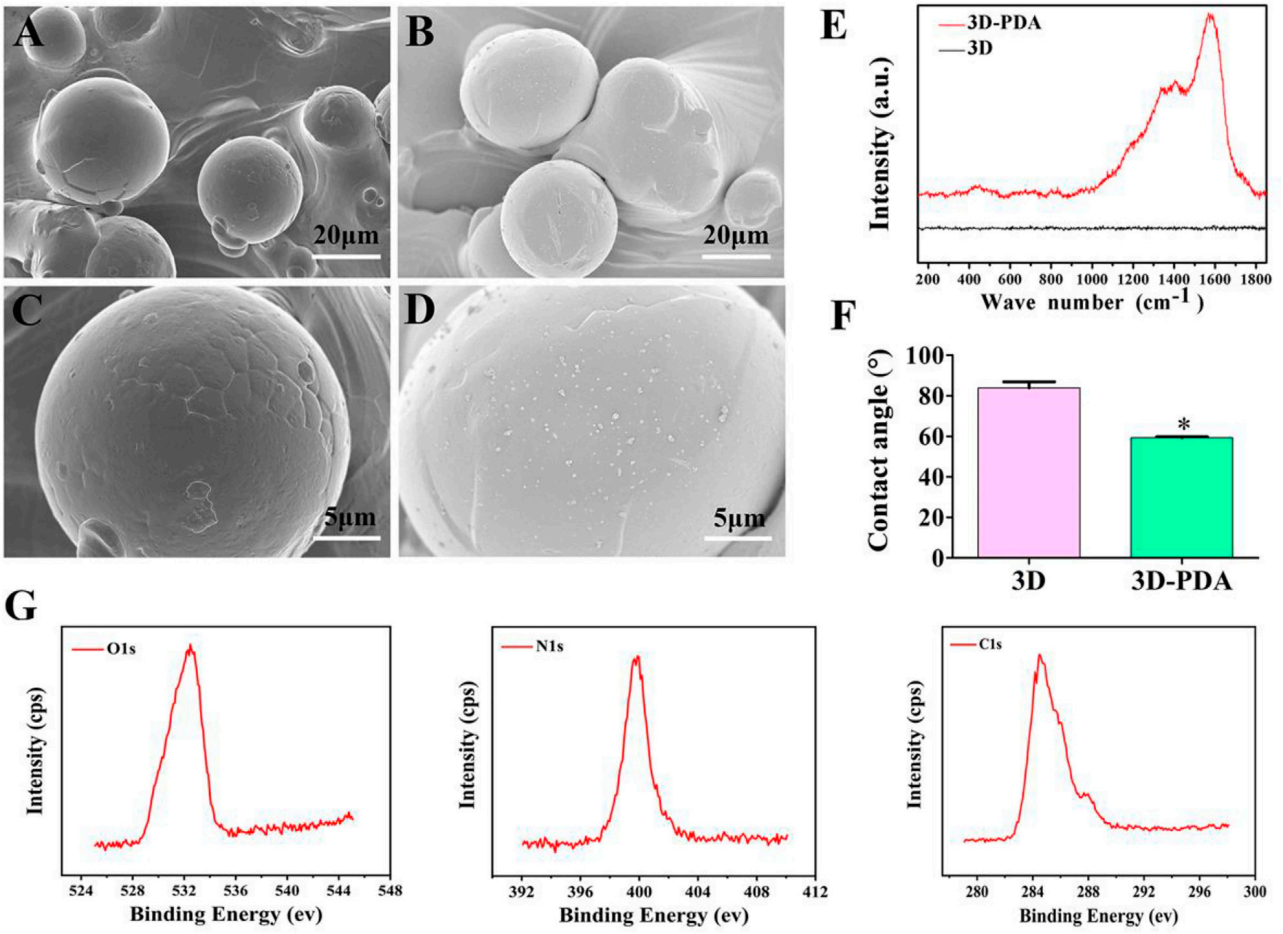
SEM images of 3D **(A,C)** and 3D-PDA **(B,D)** at low **(A,B)** and high magnifications **(C,D)**. Raman spectra analysis **(E)**, water contact angle **(F)** of 3D and Q15 3D-PDA group surfaces, and X-ray photoelectron spectroscopy spectra of the 3D-PDA surface **(G)**. ^*^ *p* < 0.05.

Figures 1A–D show the topography of the 3D and 3D-PDA sample surfaces. Both of them exhibited similar micro-scale surface morphology at low magnification. Briefly, spherical Ti6Al4V particles with sizes from 20 to 50 μm were distributed disorderly and fused on the relatively flat substrate. More importantly, granular PDA aggregates with nanometer size were evenly deposited and dispersed on the 3D-PDA samples, which was demonstrated clearly at high magnification (Figure 1D). The Raman spectra (Figure 1E) images showed that the 3D-PDA group showed the typical peaks of polydopamine at approximately 1,350 and 1,580 cm^−1^ in contrast to the 3D group. The results proved that PDA coating was fabricated on the 3D sample surface successfully. As measured by spectroscopic ellipsometer, the PDA coating thickness was approximately 30 nm.

Surface free energy (SFE) dominates the interplay between the surface, aqueous biological environment, and proteins. What is more, SFE can be reflected by water contact angle (WCA) measurements. In general, based on a WCA >90° or <90°, a surface could be classified as hydrophobic (low SFE) or hydrophilic (high SFE). And at WCA >150° or <5°, the surface could be further defined as super-hydrophilic and super-hydrophobic (Wei et al., 2020; Han and Gong 2021; Zhang et al., 2021). As presented in Figure 1F, the water contact angle of 3D-PDA was obviously lower than that of the 3D group, which suggested that the surface of the 3D-PDA group had better hydrophilicity and higher SFE. Previous research finds that PDA contains many amino and hydroxyl aqueous functional groups, which can provide a hydrophobic surface with a hydrophilic group to improve the hydrophilicity of the biological materials (Ku et al., 2010; Li et al., 2019). The hydrophilic surface is beneficial to the adhesion of extracellular matrix proteins and more RGD (R-arginine, G-glycine, D-aspartic acid) sequence sitesexposure, which has an essential role in osteoblast adhesion, propagation, and differentiation. As the XPS results showed (Figure 1G), the element peaks of O1s, N1s, and C1s were detected on the surface of the 3D-PDA group, which showed that the PDA coating was deposited successfully.

### 3.2 The Influence of a PDA-Coated Surface on the Adhesion of BMSCs

**FIGURE 2:**
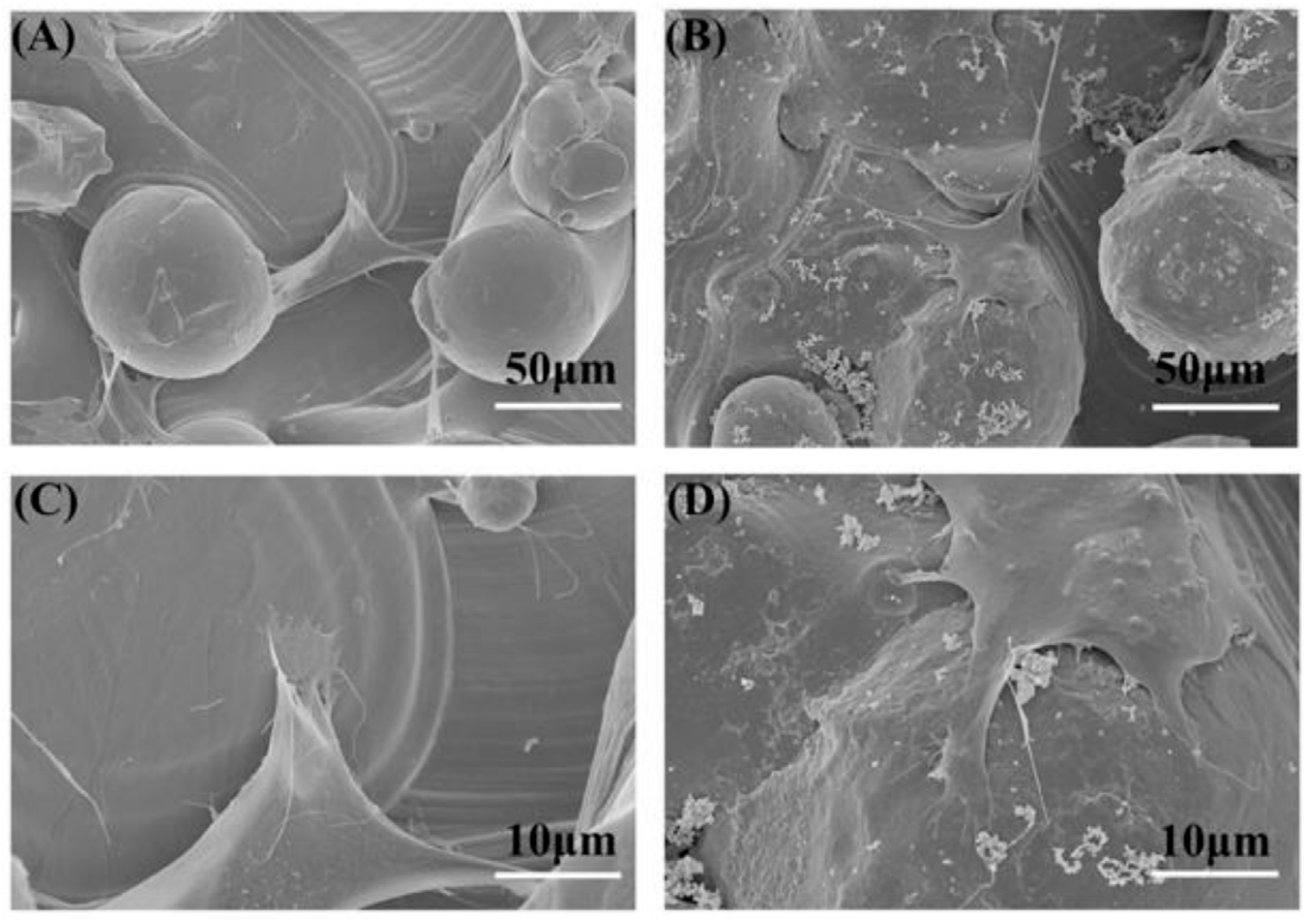
SEM images of adherent cells on the 3D **(A,C)** and 3D-PDA **(B,D)** samples after 24 h of culture at low **(A,B)** and high **(C,D)** magnifications.

The SEM results show the effect of the PDA-coated surface on the adhesion of BMSCs after seeding for 24 h (Figure 2). Both BMSCs on the two kinds of samples could grow and extend toward the three-dimensional direction. Moreover, BMSCs also could adhere from one particle to another or the substrate. Compared to the 3D group, BMSCs on the 3D-PDA sample surface spread well and had plentiful filopodia and lamellipodia, which suggested that the 3D-printed titanium surface with a PDA coating could facilitate cell adhesion. It is well known that the implant surface properties, for example, topography, wettability, and charge, could impact the conformation and orientation of the cell adhesive proteins. Then the complex cell adhesion process, including cell attachment, cell spread, cytoskeleton organization, and so on could be changed, further triggering the subsequent cell response cascades (Lebaron and Athanasiou 2000). Therefore, favorable cell adhesion is beneficial to cell survival, proliferation, differentiation, and osseointegration *in vivo*.

### 3.3 The Impact of the PDA-Coated Surface on the Propagation and Osteogenic Differentiation of BMSCs

**FIGURE 3:**
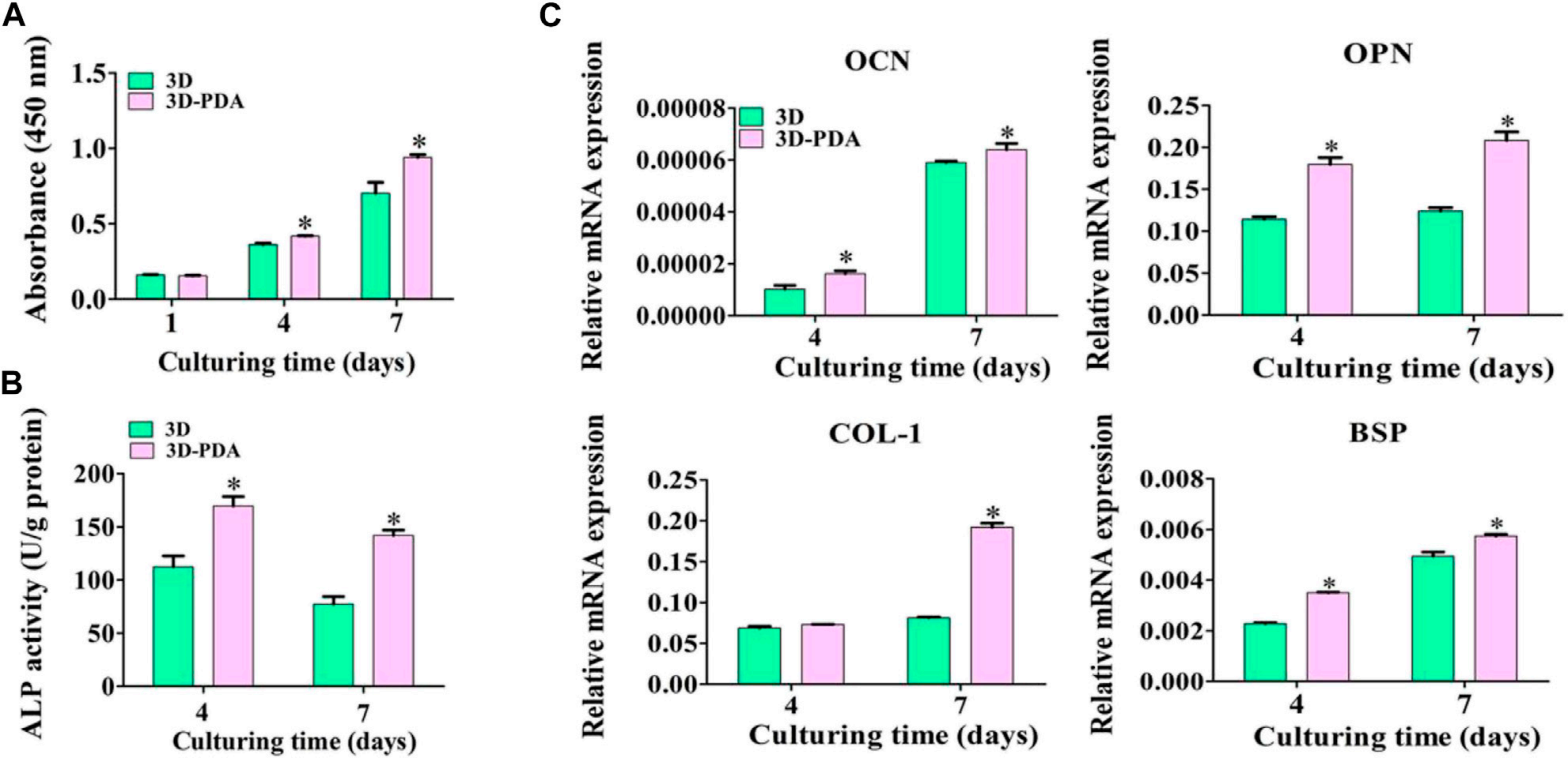
Propagation of BMSCs on the 3D and 3D-PDA samples by CCK-8 test after seeding for 1, 4, and 7 days **(A)**. ALP activity **(B)** and RT-PCR analysis of the osteogenic gene expression levels **(C)** after culturing BMSCs for 4 and 7 days on the 3D and 3D-PDA group surfaces. *p < 0.05.

The proliferation of BMSCs cultured on 3D and 3D-PDA samples were detected by the CCK-8 test (Figure 3A). There was no significant distinction between the two groups at day 1. However, the 3D-PDA samples showed higher proliferative activity than the 3D group with the increase of culturing time to 4 and 7 days. Higher cell propagation leads to increased cell colonization on the implant surface and enhances cell-cell communications. The cell-cell communications play a dramatic role in responding to external stimulus, regulating cell function development and osteoblast differentiation, resulting in larger lumps of bone tissue around the implant (Civitelli 2008; Zhao et al., 2010).

After being cultivated for 4 and 7 days, the ALP activity of BMSCs cultured on the two kinds of specimens was evaluated (Figure 3B). At days 4 and 7, BMSCs incubated on the 3D-PDA surface presented prominent ALP activity. It has been illuminated that ALP activity is an early indicator of BMSCs differentiation into osteoblasts and osteogenesis (Birmingham et al., 2012). Hence, the 3D-printed titanium implant surface with a PDA coating could promote BMSCs osteogenic differentiation. The osteogenic gene expression levels of BMSCs were detected by using RT-PCR, and the results are shown in Figure 3C. Despite the fact that the COL-1 expression level of BMSCs cultivated on 3D and 3D-PDA samples presented no significant difference at day 4, all of the OCN, OPN, and BSP expression levels of BMSCs on the 3D-PDA group were dramatically higher than that on the 3D group at each timepoint. At the process of BMSCs differentiated into mature osteoblasts, the osteogenic gene expression and local factor production were intricately regulated. At the early stage of BMSCs osteogenic differentiation, the OPN and BSP facilitated osteoprogenitor cell adhesion to the mineralized extracellular matrix and the hydroxyapatite deposition, which takes an important role in the initiation of bone formation and mineralization. COL-1 is the primary component of collagen extra cell matrix, which indicates the deposition and maturation of the bone matrix (Valenti et al., 2008). At the late stage of BMSCs osteogenic differentiation, OCN showed that premature osteoblasts developed into mature osteoblasts and bone matrix mineralization increased (Lin et al., 2013). As the above results suggested, the 3D-printed Ti6Al4V implants with a PDA coating might stimulate the osteogenic differentiation of BMSCs.

### 3.4 *In Vivo* Study

**FIGURE 4:**
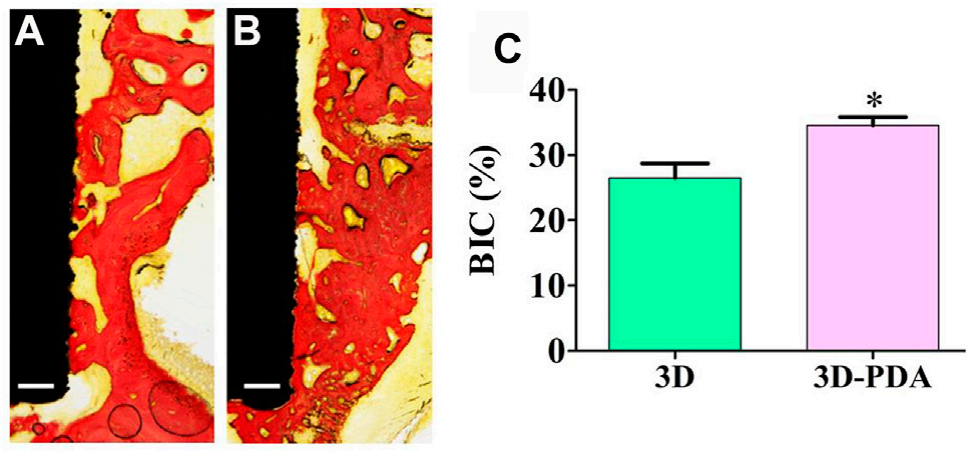
Histological images of hard-tissue slicing of 3D **(A)** and 3D-PDA **(B)** implants after implantation at week 4, scale bar = 200 μm, histomorphometry quantitive analysis of BIC percentages on implant surfaces **(C)**; *p < 0.05.

The *in vivo* osseointegration performance of 3D and 3D-PDA implants was studied by implanting them into rat femoral condyles for 4 weeks. As shown in Figure 4, compared to the 3D group, a large number of new bone tissues formed and deposited directly on the interface between the 3D-PDA implant and host bone (Figures 4A,B). And the histomorphometry quantification analysis of BIC percentages on the different implant surfaces further demonstrated that 3D-PDA had a significantly higher BIC percentage than that of the 3D group (Figure 4C). The above results implied that the 3D-printed Ti6Al4V with a PDA coating owned better osseointegration ability *in vivo*.

Taking the *in vitro* and *in vivo* results into consideration together, the 3D-printed Ti6Al4V implants with a PDA coating exhibited favorable biocompatibility and osteogenic differentiation *in vitro* and osseointegration *in vivo.* The fate of implant osseointegration depends on the cell response to the implant surface. And the surface characteristics of implant materials exert a vital influence on the cell-material interactions. In the present research, the 3D-printed Ti6Al4V implant obtained superior hydrophilicity after being modified with the PDA coating. Previous research indicates that hydrophilicity plays an important role in altering cell adhesion, propagation, and osteogenic differentiation of BMSCs (Leal-Egaña et al., 2013), which was confirmed in the present study. Hence, the advantage in improving cell response to implant material surface leads to more bone matrix deposition, mineralization, and more new bone formed rapidly to stabilize the implant.

## 4. Conclusion

It is essential to improve surface characteristics, promoting the biocompatibility and osseointegration performance of personalized 3D-printed dental implants. In the present research, the 3D-printed Ti6Al4V implant surface was successfully modified with a PDA coating, which apparently increased the hydrophilicity. Furthermore, the BMSCs adhesion, propagation, and osteogenic differentiation *in vitro* and the osteointegration *in vivo* on the PDA coating-modified implant surface were dramatically enhanced. The results suggest that PDA coating might be a feasible and favorable strategy to optimize 3D-printed implant surfaces. Moreover, the study suggests a preliminary research basis for following work to immobilize bioactive factors on 3D-printed implant surfaces.

## Data Availability

The raw data supporting the conclusions of this article will be made available by the authors, without undue reservation.
